# Natural and Engineered Resistance Mechanisms in Plants against Phytoviruses

**DOI:** 10.3390/pathogens12040619

**Published:** 2023-04-19

**Authors:** Anik Majumdar, Abhishek Sharma, Rakesh Belludi

**Affiliations:** 1 Department of Plant Pathology, College of Agriculture, Punjab Agricultural University, Ludhiana 141004, Punjab, India; anikmajumdar5757@gmail.com (A.M.); rakesh-pp@pau.edu (R.B.); 2 Department of Vegetable Science, College of Horticulture and Forestry, Punjab Agricultural University, Ludhiana 141004, Punjab, India

**Keywords:** Plant viruses, natural resistance, engineered resistance, gene editing tool, resistance genes, vegetable crops

## Abstract

Plant viruses, as obligate intracellular parasites, rely exclusively on host machinery to complete their life cycle. Whether a virus is pathogenic or not depends on the balance between the mechanisms used by both plants and viruses during the intense encounter. Antiviral defence mechanisms in plants can be of two types, i.e., natural resistance and engineered resistance. Innate immunity, RNA silencing, translational repression, autophagy-mediated degradation, and resistance to virus movement are the possible natural defence mechanisms against viruses in plants, whereas engineered resistance includes pathogen-derived resistance along with gene editing technologies. The incorporation of various resistance genes through breeding programmes, along with gene editing tools such as CRISPR/Cas technologies, holds great promise in developing virus-resistant plants. In this review, different resistance mechanisms against viruses in plants along with reported resistance genes in major vegetable crops are discussed.

## 1. Introduction

Plant viruses cause significant yield loss in the cultivation of many agricultural and horticultural crops around the world. Conventional methods of virus control have been used for a long time, including crop rotation, other types of cultivation, removal of infected plant debris, and chemical control of insect vectors. Since these methods have very little impact in the mitigation of virus diseases, numerous inventive methods to manage viral infections in plants have been developed as a result of a growing understanding of the molecular genetics of plant viruses as well as of the hosts’ defence mechanisms. The primary defence mechanisms against viruses in plants include innate immunity, RNA silencing, translational repression, dominant resistance genes, and autophagy-mediated degradation [1]. The rise of gene editing technologies has also enabled researchers/scientists to engineer artificial resistance against viruses in plants. How do different antiviral mechanisms in plants operate to limit the successful pathogenesis of viruses? In this review, we summarise the recent advances in the plant defence mechanisms against phytoviruses along with some reported virus resistance genes in major vegetable crops.

## 2. Virus Infection Cycle within the Plants

The virus infection cycle within the plants can be narrated as described by Calil and Fontes [2]. Viruses enter plants through wounds created mechanically, vectors, pollen, seeds or vegetative propagation, pinocytosis, fungal parasites, and epidermal hairs. After entry, the uncoating of the virus capsid takes place in the cytoplasm. At this stage, the translation of viral proteins occurs using host translation machinery. Translation events are further divided into two parts, i.e., early translation (synthesis of proteins required for viral replication, e.g., RNA-dependent RNA polymerase) and late translation (synthesis of coat proteins). Using these replication-associated proteins, virus genome replication occurs either in the cytoplasm or in the nucleus using different mechanisms. The progeny genomic strands are then encapsidated with newly synthesised coat proteins, leading to numerous progeny virion particles. These progeny virion particles move from one cell to another via plasmodesmata and/or long-distance movement occurs through phloem with the help of virus movement proteins (MP). Finally, they get released outside the host cell and transmitted to new hosts by means of different mechanisms, and this cycle of infection continues.

**Figure 1:**
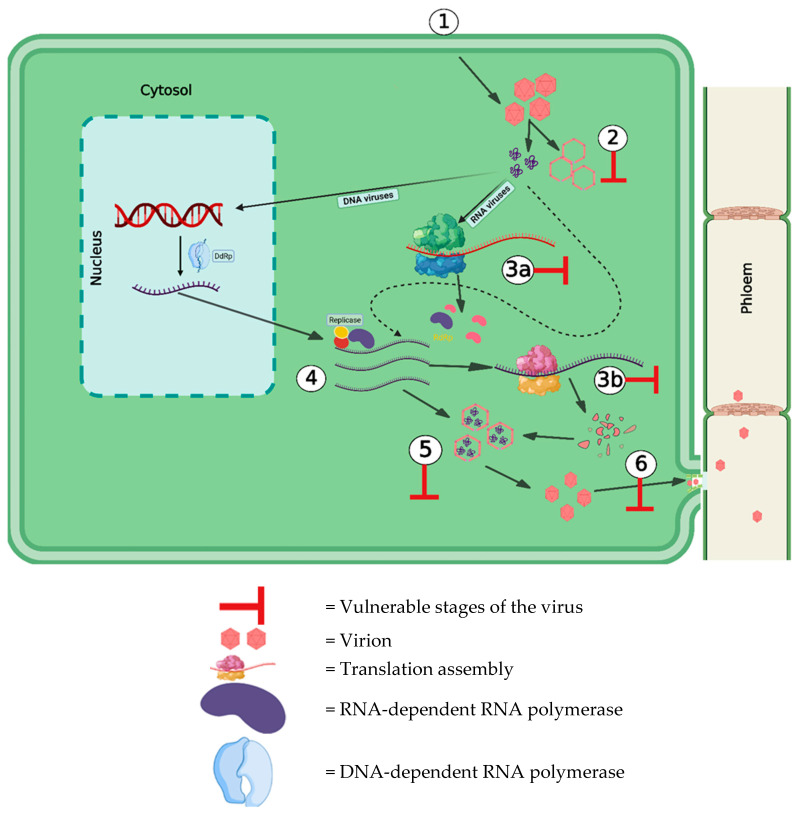
Possible ‘weak points’ in the virus infection cycle targeted by host defences: (**1**) Entry into plant cell through vector/pinocytosis/pollen/seed/vegetative propagation/epidermal hair/fungal parasite. (**2**) Uncoating of viral capsid. (**3a**) Early translation. (**3b**) Late translation. (**4**) Genome replication. (**5**) Encapsidation. (**6**) Cell-to-cell movement through plasmodesmata. (Figure created in Biorender.com, accessed on 8 March 2023).

This infection process is targeted by several host defence systems which help the host to counter the virus attack. There are several points in the infection cycle which can be targeted by host defences such as the uncoating of the virus capsid, translation events, encapsidation, and cell-to-cell movement (Figure 1).

## 3. Antiviral Defence Mechanisms in Plants

This can be divided into two types: Natural resistance to plant viruses and Engineered resistance to plant viruses.

### 3.1. Natural Resistance to Plant Viruses

Possible natural resistance mechanisms against viruses include innate antiviral immunity, translation repression, small RNA-mediated antiviral defence, dominant viral resistance genes, resistance to virus movement, autophagy, and cross protection.

#### 3.1.1. Innate Antiviral Immunity

**Figure 2:**
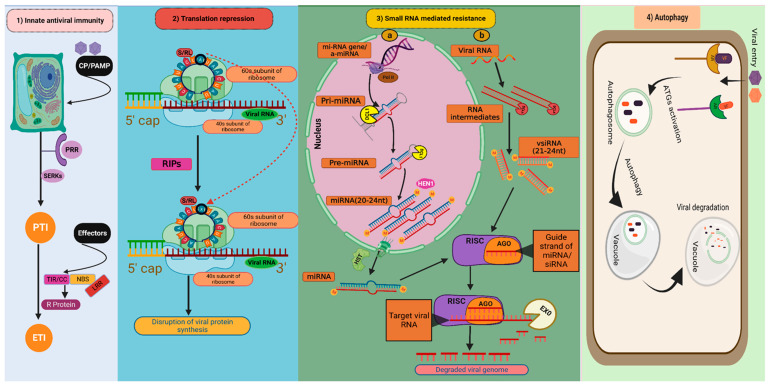
Natural antiviral defence mechanisms in plants: (**1**) Innate antiviral immunity; (**2**) Translation repression; (**3**) Small RNA-mediated resistance: (**3a**) MicroRNA mediated resistance. (**3b**) Small interfering RNA-mediated resistance. (**4**) Autophagy (Figure created in Biorender.com, accessed on 8 March 2023). CP/PAMP: Coat protein of virus/Pathogen-associated molecular patterns; PRR: Pattern recognition receptors; SERK: Somatic embryogenesis receptor kinases; PTI: PAMP triggered immunity; ETI: Effector-triggered immunity; S/RL: Sarcin/ricin loop; RIPs: Ribosome-inactivating proteins; 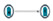: depurination of S/R loop; miRNA gene/amiRNA: Micro RNA gene/artificial micro RNA; Pri-miRNA: Primary micro RNA; Pre-miRNA: Precursor micro RNA; miRNA: micro RNA; vsiRNA: Virus-derived small interfering RNA; DCL1: DICER LIKE 1; DCLs: DICER LIKE proteins; HST: HASTY; HEN1: HUA ENHANCER 1; RISK: RNA-induced silencing complex; AGO: Argonautes; EXO: Exonuclease; AR: Autophagy receptors; VF: Viral factors; ATGs: Autophagy-related genes.

There are two layers of plant immune responses against microbial pathogens, i.e., PAMP (pathogen-associated molecular pattern) triggered immunity (PTI) and Effector-triggered immunity (ETI) (Figure 2 (**1**)). PTI is the initial mechanism by which plants detect the microbes at the cell membrane through detection of conserved PAMPs by extracellular pattern recognition receptors (PRRs) [3]. PRRs begin to dimerize as soon as the PAMPs are detected and associate with cofactors such as somatic embryogenesis receptor-like kinases (SERKs). This sets off a cascade of intracellular signalling events, such as the generation of reactive oxygen species (ROS), ion influx, increase in the production of defence hormones, and mitogen-activated protein kinases (MAPKs) activation. All these events result in the expression of the pathogenesis-related (PR) proteins, synthesis and deposition of callose at the plasmodesmata, and cell wall strengthening, leading to the generation of resistance response. [4]. Activation of PTI sometimes leads to hypersensitive response (HR) causing programmed cell death (PCD) that causes necrotic spots at the infection site [5]. Although plant PTI against other phytopathogens is well understood, plant viruses are traditionally known as non-PAMP coding pathogens [6]. However, some evidence implies that PTI also has a significant influence on both susceptible and resistant plant-virus interactions. For instance, exogenous application of double-strand RNA (dsRNA) caused SERK-1-dependent PTI responses in *Arabidopsis* [7]. In another instance, tobacco and *Arabidopsis* showed PTI-like responses due to the coat proteins of tobacco mosaic virus and potato virus X, respectively [8,9].

Pathogens in response to PTI introduce particular proteins called effectors in plant cells to weaken PTI-mediated defence. In response to these effectors, host plants depend on specific intracellular receptors known as R gene proteins which cause direct or indirect identification of the pathogen effector molecules. These R gene products can block the effectors and activate the effector-triggered immunity (ETI) [10]. R genes mediating resistance against various plant viruses have been extensively cloned over the last decade due to their apparent practical value. Functional R proteins are composed of three domains: a central nucleotide-binding site (NBS) domain, a leucine-rich repeat (LRR) domain, and an N terminal Toll Interleukin-1 receptor (TIR) or coiled-coil (CC) domain. Systemic acquired resistance (SAR) is a condition that can occur as a result of both PTI and ETI and is characterised by the development of resistance in the tissues that are distal to the infection [11]. While the non-expressor of PR1 (NPR1) is a protein with ankyrin domains, which is necessary for triggering salicylic acid (SA) signalling and establishing SAR, salicylic acid is the principal plant hormone responsible for establishing SAR [12]. SAR, together with pathogenesis-related proteins, confers resistance to the host plants against various pathogens [13].

#### 3.1.2. Translation Repression as Virus Resistance

The initiation of translation is a critical step in protein synthesis that demands an array of eukaryotic initiation factors (eIFs). The translation of eukaryotic mRNA hinges on the association between the translation eukaryotic initiation factor 4E (eIF4E) and their 5′ m7 G cap structure, and the 3′ polyA tail’s interaction with the polyA-binding protein (PABP) also heightens the process. Although translation factors are not typically encoded by viruses, they have developed several techniques to hijack translation factors from their hosts, resulting in the promotion of viral RNA translation while compromising the translation of endogenous mRNAs. Mutated isoforms of the translation IFs, i.e., eIF4E and eIF4G have been found linked to many plant recessive resistance genes. These mutations typically prevent host factors from interacting with viral RNAs or proteins to suppress viral protein translation, an endogenous antiviral mechanism that has emerged recently [14]. Recessive resistance occurs when a component (or components) of the translation machinery recruited by viruses ceases to operate, resulting in a ‘loss of susceptibility’ to viruses. For instance, a family of proteins known as ribosome-inactivating proteins (RIPs) can inhibit the synthesis of new proteins by depurinating the sarcin/ricin loop (SRL) of rRNA [15] (Figure 2 (**2**)). The most thoroughly studied RIP with antiviral activity is the pokeweed antiviral protein (PAP) from *Phytolacca americana*. PAP slows down the spread of various plant viruses, including the cauliflower mosaic virus, potato virus X, and cucumber mosaic virus [16]. In the interaction between plants and viruses, small RNA-related translation suppression can be quite significant. For instance, in tomato plants infected with the tomato ringspot virus, recovery of symptoms is associated with the AGO1-dependent translation repression of viral RNA2. [17]. Additionally, the inability of one strain of plum pox virus to recruit translation initiation factors conferred resistance against this strain in wild-type *Arabidopsis thaliana* and *Chenopodium foetidum* [18].

#### 3.1.3. Small RNA-Mediated Antiviral Defence

Small RNAs (sRNA) are essential for the epigenetic and post-transcriptional control of gene expression in plants throughout growth, developmental, and biotic/abiotic stress responses. Recent research has highlighted the role of two distinct families of small RNAs, known as small interfering RNA (siRNA) and micro RNA (miRNA) with respect to biotic stress responses in plants. By regulating the gene expression of the modulators of host defence pathways, these sRNAs activate antiviral defence during virus infection. RNA silencing, commonly referred to as RNA interference (RNAi), is a conserved evolutionary method for regulating endogenous expressions of genes and preventing the entry of foreign nucleic acids such as viruses and transposons [19]. The plant type III endoribonucleases or dicer-like (DCL) proteins detect and cleave the virus-derived double-stranded RNA (dsRNA) into small 20–24 nucleotide RNA duplexes known as virus-derived short interfering small RNAs (vsiRNAs) [20]. The vsiRNAs incorporated into argonaute proteins (AGOs) form the core component of the RNA-induced silencing complex (RISC), which cleaves homologous viral RNAs and/or suppresses translation of viral protein synthesis [21,22] (Figure 2 (**3b**))

Unlike siRNAs, miRNAs are endogenously produced non-coding short RNAs by RNA polymerase II from ssRNA precursors with a hairpin structure. [23]. Along with the well-established function of vsiRNAs, research findings suggest that miRNAs play a significant role in plant antiviral defence. At the initiation of miRNA synthesis, a primary miRNA transcript, or Pri-miRNA is synthesised after transcription and is made up of an incomplete stem-like structure of 100–120 nucleotides. Then, the precursor miRNA (Pre-miRNA) which is about 70 nucleotides long, formed after processing of Pri-mRNA by the DCL1 complex in the nucleus. Finally, the cleaving of 20–24 nucleotides from the initial cleavage point results in the formation and release of the miRNA/miRNA duplex from the stem-like structure. HUA Enhancer 1 (HEN1) methylates miRNA-miRNA duplex to protect it from degradation in the cytosol. Finally, they are transported to the cytosol by exportin-5 homolog HASTY (HST). In the cytosol, mature miRNAs are incorporated into RISC complexes having AGO proteins. The majority of the animal miRNAs that have been studied so far have miRNA and mRNA base pairing, which hinders the target mRNA translation. This is one of two ways that miRNAs guide RISC to down-regulate target mRNAs. Contrarily, the majority of plant miRNAs form substantial base pairing and directly cleave their target mRNAs. [24] (Figure 2 (**3a**)). Recently, the roles of various miRNAs, such as miR168, miR528, miR319, and miR444 in rice antiviral immunity have been identified. By modifying jasmonic acid (JA) signalling, MiR319 has been reported to confer antiviral resistance to rice against the rice-ragged stunt virus and to wheat against the rice black-streaked dwarf virus. [25].

#### 3.1.4. Dominant Viral Resistance Genes

A few dominant resistance genes that function independently of the traditional innate immune signalling pathway have also been discovered in the past ten years. These dominant resistance genes express proteins that cannot be incorporated into the so-called plant innate immunity because they differ structurally from conventional R proteins. The majority of these dominant resistance genes inhibit the action of viral proteins by interacting with them in a direct manner. These dominant resistance gene products are referred to as atypical dominant viral resistance proteins (ADVRPs). The majority of ADVRPs that have been identified so far are from a protein family called lectin. Restricted TEV movement (RTM) 1, a member of the lectin-protein family is found in *Arabidopsis*, and it particularly provides resistance to a number of potyviruses, including TEV, lettuce mosaic virus (LMV), and plum pox virus by inhibiting their long-distance movement [26]. A lectin-like ADVRP of *Arabidopsis* called Jacaline-type lectin required for potexvirus resistance 1 (JAX1) imparts broad spectrum resistance to potexviruses at the initial infection stage by suppressing the activity of viral RNA-dependent RNA polymerase (RdRp) [27]. BanLec-1, another lectin from *Musa paradisiaca*, attaches to the TMV CP and inhibits virus infection in plants [28].

#### 3.1.5. Resistance to Virus Movement within and between the Cells

For plant viruses to infect the entire plant system, they must spread from the initially infected cells to nearby ones. This must occur after the virus multiplication has begun within the cytoplasm and/or nucleus of a plant cell in a susceptible host. Host resistance to viral infection is evident when the virus only appears to affect one or a small number of cells but is unable to move past this initial focus of infection. At this point, resistance can be brought on by active host defence systems that swiftly thwart virus spread or by a breakdown in the connections between plant and viral components necessary for cell-to-cell movement. It is known that a variety of host gene alterations hinder the cell-to-cell movement of plant viruses. The *cum1* and *cum2* mutations in *Arabidopsis* cause reduced multiplication of CMV, thus rendering its movement in adjacent cells [29]. *Pvr11* and *Sbm1* were found to be mutated at a locus expressing eIF4E in pepper and pea, respectively [30,31]. eIF4E is thought to play a role in viral RNA replication or translation, although it may also be involved in cell-to-cell movement. The restricted-TEV-movement (RTM) genes *RTM1*, *RTM2*, and *RTM3* prevent systemic tobacco etch virus (TEV) movement between plant cells. These genes interact with the virus CP and are expressed in phloem sieve elements [32]. Similar to this, BTR1 is a ribonucleoprotein K-homology RNA binding protein that binds to ToMV (tomato mosaic virus) genomic RNA and limits its movement between cells [33]. By inducing cell death at the sites of infection and restricting the movement between cells, the *Ny-1* gene confers potato virus Y (PVY) resistance in potatoes [34].

#### 3.1.6. Autophagy as Antiviral Mechanism against Plant Viruses

Proteins and defective organelles are transferred to vacuoles or lysosomes for destruction through autophagy, which is an evolutionarily conserved intracellular degradation mechanism [35]. Autophagy also contributes to antiviral defence mechanisms in plants. Virus particles upon entry are recognised by specific autophagy receptors (AR). After recognition, autophagy-related genes (*ATGs*) get activated and initiate the formation of autophagosome, which ultimately fuse with the vacuole leading to the vacuolar degradation of autophagosome contents which also harbour viral particles (Figure 2 (**4**)). As TMV aggregates at infection sites in *Beclin-1* or *ATG7* silenced plants, it has been hypothesised that autophagy contributes to plant defence against viruses. It is interesting to note that induction of autophagy extends beyond the TMV infection sites to uninfected neighbouring tissues, where it prevents cell death [36]. Several reports have recently emphasised the significance of this mechanism in restricting virus multiplication. To prevent the replication of the cotton leaf curl Multan virus (CLCuMuV), *ATG8* specifically interacts with the beta-satellite (βC1) of the CLCuMuV [37]. Not only DNA viruses but RNA viruses are also targeted by the antiviral effects of autophagy. Nuclear inclusion protein B (NIb) of turnip mosaic virus (TuMV), interacts with autophagy-related gene 6 (*ATG6*, also known as *Beclin1*) to prevent viral replication [38]. Additionally, it was reported that TuMV infection triggered autophagy, which reduced viral RNA accumulation in *Arabidopsis*. [39].

#### 3.1.7. Cross Protection

Cross protection has been utilised to manage viral diseases together with protein and RNA-mediated resistance. By infecting the host plant with a mild strain of the virus, the resistance is conferred effectively. The infection caused by attenuated strain makes the affected plant immune to further infections by a viral strain that is closely related to the inoculated virus. After the attenuated strain has been inoculated, it may trigger strain or sequence-specific resistance against the challenger virus. In a number of experiments, the effect of coat protein on cross-protection reactions has been examined. When systemically expressed using PVX as a virus vector, various TMV CP mutants with altered CP aggregation demonstrated that the CP mutants with high assembly capacity offered effective cross protection against TMV infections. TMV cross protection and CP-mediated resistance may therefore depend on CP’s capacity to prevent the challenging virus from uncoating its capsid [40]. Numerous researchers have also discovered that mutations to virus silencing suppressors can reduce the severity of symptoms. For instance, mutations in the 126 kD replicase, a suppressor of silencing from the pepper mild mottle virus (PMMoV), resulted in milder symptoms as well as offering pepper plants resistance through cross protection [41].

### 3.2. Engineered Resistance to Plant Viruses

Engineered resistance against plant viruses can be divided into two parts: pathogen-derived resistance (PDR) and gene editing technologies.

#### 3.2.1. Pathogen-Derived Resistance

The majority of the functions played by plant viruses during the replication cycle are well-characterised since they have short genomes and few genes. Viruses are suitable targets for developing artificial resistance based on the concept of pathogen-derived resistance (PDR). Sanford and Johnston were the ones who originally introduced this idea [42]. The most popular and effective method of obtaining PDR has been the exploitation of virus CP genes. High levels of resistance in transgenic plants have been shown to result from host plants expressing the CP of a number of RNA viruses including TMV, PVX, CMV, and TRV. This suggests that CP-mediated resistance is dependent on inhibiting the disassembly of the infecting virus. A noteworthy achievement is the use of transgenic papaya expressing the CP transgenes of papaya ring spot virus for the control of papaya ringspot disease in Hawaii [43]. Some reported cases of PDR in crop plants have been enlisted in Table 1.

#### 3.2.2. Gene Editing Technologies

These are further divided into Engineering ZFN or TALEN-based resistance and CRISPR/Cas technology.

##### Engineering ZFN or TALEN-Based Resistance against Viruses

It only became possible a decade ago to manipulate the genetic material in many cellular organisms, thanks to a novel technique called genome editing. The first-generation genome editing technologies are zinc finger nucleases (ZFNs) and transcription activator-like effector nucleases (TALENs) [52]. TALENs and ZFNs are chimeric proteins formed by joining the nonspecific cleavage domain of the enzyme FokI to the DNA binding domain (DBD) from either a transcription activator-like effector or a zinc finger protein, respectively. The cleavage domain cuts the DNA to create double-strand breaks (DSB) at the target site once the DBD detects a particular recognition sequence in the target DNA. In addition to integrating, deleting, or mutating desired genes, these genome editing techniques also give a potent weapon in the armoury against plant viruses. An artificial zinc finger protein (AZP) was designed to target the intergenic region (IR) of the beet-severe curly top virus (BSCTV) in *Arabidopsis* [53]. This protein differs from ZFN in that it lacks the cleavage domain. The stem-loop structure found in the geminiviruses IR is critical for virus replication, which initiates with the binding of viral replication initiation protein (Rep) [54]. The BSCTV IR is efficiently bound by the transgenically produced AZP, prevents Rep binding and thereby inhibits virus infection. ZFN technology has been reported to target the *Rep* genes tobacco curly shoot virus (TbCSV) and tomato yellow leaf curl China virus (TYLCCNV) in tobacco plants, which showed a considerable suppression of viral genome replication [55].

##### Engineering CRISPR/CAS-Based Resistance against Plant Viruses

**Figure 3:**
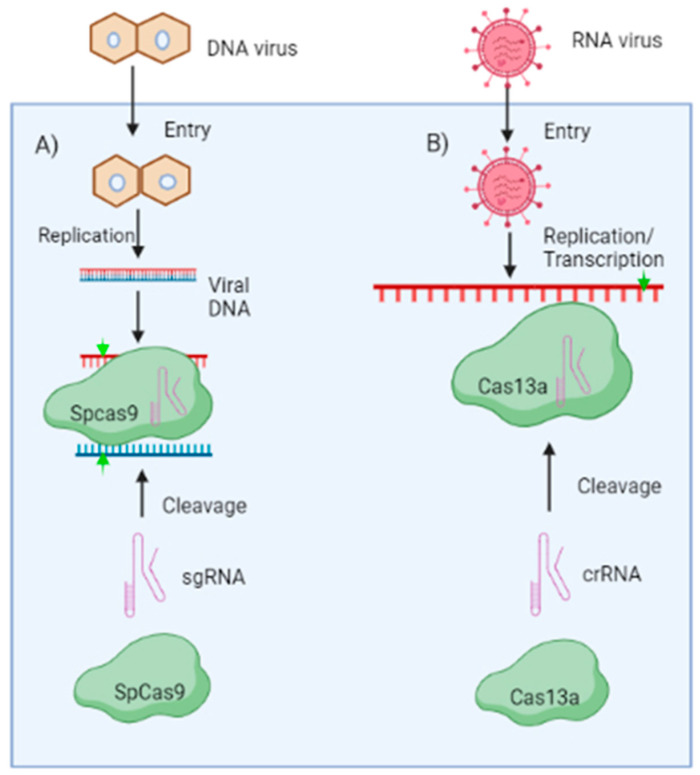
CRISPR/Cas-based engineered resistance mechanisms: The Cas protein and sgRNA make up the CRISPR system. The virus-targeting Cas protein and its cognate transgenic or transit expression can successfully inhibit virus infection. (**A**) When a DNA virus enters a plant cell, its genome is converted into dsDNA intermediate that is targeted by the Cas9 protein from *Streptococcus pyrogenes* (SpCas9). (**B**) While it has been demonstrated that Cas13a effectively confers resistance to RNA viruses. Upon entry, Cas13a can cleave/bind the virus genome under the guidance of their associated sgRNA or crRNA, respectively. The cleave sites on viral mRNA are shown by arrowheads in green. (Figure created in Biorender.com, accessed on 8 March 2023).

A promising tool for plant genome modification is the CRISPR (clustered regularly interspaced palindromic repeats)/CRISPR associated 9 (CRISPR/Cas9) system. The Cas9 nuclease and a single guide RNA (sgRNA) with a 20-nucleotide-long spacer sequence which guides the Cas protein to the DNA or RNA target make up the CRISPR/Cas machinery. Recently, various research has revealed the generation of virus-resistant plants with CRISPR/Cas9 technique having resistance durability [56]. By disrupting viral genes rather than silencing them at the RNA level, CRISPR/Cas9 technique provides an extra benefit to RNA interference (RNAi) or artificial microRNAs (amiRNAs) for generating virus-resistant plants [57]. The first system to be used as a platform for genetic engineering was the Class II type II CRISPR/Cas9 technology, having a single Cas9 nuclease. The Cas9 is directed by two small RNAs, transactivating CRISPR RNA (tracrRNA) and CRISPR RNA (crRNA). sgRNA is generated by the fusion of the tracrRNA and crRNA, which guides Cas9 to identify and degrade the target sequence [58] (Figure 3). This two-component system’s effectiveness and simplicity allow for developing virus resistance in different plant genera (Table 2).

##### CRISPR/CAS-Based Resistance against DNA Viruses

DNA is the target molecule for the CRISPR/Cas9 technology derived from *Streptococcus pyrogenes* (Figure 3A). In order to inhibit the geminiviruses, the CRISPR/Cas9 technology was initially used to target its genomic DNA during the replication stage. It was demonstrated that numerous geminiviruses might be simultaneously targeted by a sgRNA, which targets the conserved sequence (TAATATTAC) in the IR, which is an essential site for replication initiation [59]. In tobacco and *Arabidopsis*, CRISPR/Cas9 was successfully used by three different research teams to develop geminivirus resistance. sgRNAs that specifically target the IR, Rep, or CP loci were designed, and these greatly diminished geminivirus disease symptoms in plants [59,60,61]. To provide resistance to the sense and anti-sense regions of the tomato yellow leaf curl virus (TYLCV), CRISPR/Cas9 cassettes were developed for *Nicotiana benthamiana*. It is noteworthy that the virus was hindered by the CRISPR/Cas9 system, which also significantly lowered viral titre in leaves and caused reduced viral symptoms. The virus genome was modified by CRISPR/Cas9 through the error-prone non-homologous end joining repair (NHEJ) after it targeted the viral dsDNA for cleavage [62]. A similar technique was recently employed in barley as well, establishing highly effective resistance against the wheat dwarf virus (WDV). In order to develop resistance against multiple virus strains, the genome sequences of two barley and two wheat WDV strains were examined to identify potential sgRNA target sites situated in conserved regions. Transgenic barley lines harbouring sgRNA constructs 1,3 and 4 exhibited no symptoms and the presence of the virus was neither detected by northern blotting nor by polymerised chain reaction (PCR) [63]. CRISPR/Cas9 has also been found efficient in reducing the virulence of the cauliflower mosaic virus in *Arabidopsis*. It was reported that *Arabidopsis* transgenic plants involve the expression of several sgRNAs that target the CaMV CP coding sequences. Moreover, it was found that siRNAs with 21–24 nucleotides (nt) were produced from sgRNAs, most of which were mapped to 3’ end of the sgRNA backbone region and less frequently to the spacer region, which binds to the CaMV coat protein-coding sequences. This finding proved CRISPR/Cas9 effectiveness against double-stranded DNA viruses as well [64].

##### CRISPR/CAS-Based Resistance against RNA Viruses

Many viruses that infect both humans and plants have RNA genomes, which lack any DNA intermediates in their life cycle. As a result, they are immune to the traditional DNA targeting CRISPR/Cas9 mechanism. *Francisella novicida*, a pathogenic bacterium, whose FnCas9 orthologue targets RNA has been exploited to neutralise various human and/or plant viruses. Numerous Cas protein variants, including the Cas9 from *Francisella novicida* (FnCas9) and Cas13a from *Leptotrichia shahii* (LshCas13a) or *Leptotrichia wadei* (LwaCas13a), have been reported to target RNA in vivo, which shows promising results against RNA viruses (Figure 3B). For instance, CMV and TMV were the targets for modified FnCas9 and its sgRNA, in tobacco and *Arabidopsis* having the antiviral mechanisms, respectively. It caused lesser virus accumulation and resulted in reduced symptoms in tobacco and *Arabidopsis* [65]. It is interesting to note that virus inhibition by FnCas9 requires RNA-binding abilities rather than its cleavage mechanism. The LshCas13a system was designed to destroy the genomic RNA of southern rice black-streaked dwarf virus (SRBSDV) and rice stripe mosaic virus (RSMV) in rice, as well as to cleave the genomic RNA of TMV in tobacco [66]. Although these systems might result in non-specific RNA cleavage, RNA genomes as target sites are preferable because they would not cause any heritable off-target effects on the host genomic DNA. Furthermore, the RNAi system of plants will destroy the ‘cleaved off’ genomic RNAs, thus giving RNA viruses very less chance to avoid the CRISPR/Cas system.

**Table 2:** CRISPR/Cas-mediated resistance against viruses in different plant genera. Cas9 obtained from *Streptococcus pyrogenes* (SpCas9), FnCas9 from *Francisella novicida*, and Cas13a from *Leptotrichia shahii* (LshCas13a).

CRISPR/Cas System	Host Plant	Virus	References
SpCas9	Tobacco and *Arabidopsis*	Beet-severe curly top virus, Beet curly top virus	[59,61]
	Tobacco	Bean yellow dwarf virus	[60]
	Barley	Wheat dwarf virus	[63,64]
FnCas9	Tobacco	Tobacco mosaic virus	[65]
	Tobacco and *Arabidopsis*	Cucumber mosaic virus	[65]
	Tobacco	Tobacco mosaic virus	[66]
LshCas13a	Rice	Southern rice blacked streaked dwarf virus	[66]
	Tobacco	Turnip mosaic virus	[67]

## 4. Reported Resistance Genes (Dominant and/or Recessive) against Plant Viruses in Major Vegetable Crops

Among plant pathogens, viruses are known to cause major yield losses to most of the significant field and horticultural crops around the world. As a result, considerable effort has been put into breeding for virus resistance. The most recent developments in molecular biology techniques have made it possible to engineer virus resistance in plants. The initial step in a resistant breeding programme is to identify a resistance source of the specific virus. Thereafter, the breeding strategy for a crop is chosen to keep in view the biology of its reproduction, type of cultivar, and inheritance of the resistance (i.e., monogenic; oligogenic or polygenic; dominant or recessive).

It was found that around 80% of the host-virus combinations have monogenic resistance, with the remaining 20% being either oligo- or polygenic [68]. Genes that confer resistance to the virus are designated as either dominant or recessive. The nucleotide-binding site leucine-rich repeat (NBS-LRR) proteins that are coded by dominant resistance genes provide resistance by interacting with the avirulence (*Avr*) gene products of the pathogen [69]. Members of NBS-LRR proteins can be further divided based on whether they have an N terminal coiled-coil (CC) domain or a Toll interleukin-1 receptor (TIR) homology domain. The use of dominant resistance genes conferring total resistance is an interesting option for plant breeders and they have been used widely in the breeding programme. Although the resistance activation by pathogen *Avr* determinants is dependent on precise molecular interactions, virus resistance genes appear to have more specificity than one might anticipate [70]. While affording a wider spectrum of protection than dominant resistance genes, recessive resistance is defined as incompatible interactions between a plant virus and its host caused by loss of or mutations in host components [15]. As viruses totally depend on their hosts to complete their life cycle, disruptions of host-virus interactions caused by mutations or deletions in host components essential for the virus infection cycle may result in recessive resistance. Host proteins necessary for intracellular and intercellular transport as well as virus replication will be the possible targets for recessive resistance genes [71]. In the following section, some of the reported dominant and/or recessive virus resistance genes in major vegetable crops have been enlisted (Table 3, Table 4, Table 5, Table 6, Table 7 and Table 8).

## 5. Conclusions

Despite great progress over the past decade, many questions remain unanswered, such as the molecular mechanisms controlling incompatible virus-plant interactions, the existence of PAMPs from viruses and their associated PRRs, and the mechanisms controlling the mixed infection-assisted resistance loss in host plants. Increased understanding of the natural resistance mechanisms of plants against viruses has been attained through in-depth investigations on virus/plant interactions employing innovative technologies. Numerous antiviral defence strategies have also been identified by utilising the growing understanding of the molecular interactions between viruses and their host plants. The use of well characterised natural resistance genes that can be inserted by marker-assisted breeding techniques is very promising along with genome editing tools, especially CRISPR/Cas-mediated technologies which stand out for their simplicity, robustness, and versatility in developing virus-resistant plants.

## 6. Future Directions

A promising approach to building genetic resistance to viruses in plants is to use host genes with antiviral activity. However, viruses are rapidly evolving and have a remarkable ability for mutation. Although significant progress has been made over the past few years in the understanding of the structure and function of the plant-virus resistance genes and in the deployment of these genes in various fields and horticultural crops, more work still needs to be carried out. Viral disease threats are emerging, particularly in developing countries, due to the expansion of monoculturing of specific crops for which the resistance resources are still unknown. Interpretation of the techniques which are significantly promising in virus resistance, and the different pathways by which these mechanisms can be deployed in resistance breeding are definitely going to be a centre of attraction for research in the coming future.

## Figures and Tables

**Table 1:** Reported cases of pathogen-derived resistance in crops (against plant viruses).

Crop Species	Target Pathogen	Gene Expressed	Reference
*Solanum lycopersicum*	Cucumber mosaic virus	Coat protein	[44]
*Capsicum annum*	Cucumber mosaic virus	Coat protein	[45]
*Solanum tuberosum*	Potato leaf roll virus	Coat protein	[46]
*Carica papaya*	Papaya ringspot virus	Coat protein	[47]
*Prunus domestica*	Plum pox virus	Coat protein	[48]
*Phaseolus vulgaris*	Bean golden mosaic virus	+,− RNA of virus replication protein	[49]
*Carica papaya*	Papaya ringspot virus	Viral replicase gene	[50]
*Prunus domestica*	Plum pox virus	Coat protein, P1, HC-Pro	[51]

**Table 3:** Reported resistance genes against plant viruses in tomato (*Solanum lycopersicum*).

R Gene	Name of the Virus	Reference
*Ty-1*	Tomato yellow leaf curl virus	[72]
*Ty-2*	Tomato yellow leaf curl virus	[73]
*Ty-3*	Tomato yellow leaf curl virus	[74]
*Ty-4*	Tomato yellow leaf curl virus	[75]
*ty-5*	Tomato yellow leaf curl virus	[76]
*Ty-6*	Tomato yellow leaf curl virus	[77]
*Sw-5*	Tomato spotted wilt virus	[78]
*Sl5R-1*	Tomato spotted wilt virus	[79]
*SICSH3*	Tomato spotted wilt virus	[80]
*Tm-1*	Tomato mosaic virus	[81]
*Tm2* and *Tm-2*^2^	Tomato mosaic virus	[82]
*SlSw5a*	Tomato leaf curl New Delhi virus	[83]

**Table 4:** Reported resistance genes against plant viruses in potato (*Solanum tuberosum*).

R Gene	Name of the Virus	Reference
*Rx-1*	Potato virus X	[84]
*Rx-2*	Potato virus X	[85]
*Ry_adg_*	Potato virus Y	[86]
*Y-1*	Potato virus Y	[87]
*Ry_chc_*	Potato virus Y	[88]
*Ry_fsto_*	Potato virus Y^NTN^	[89]

**Table 5:** Reported resistance genes against plant viruses in chilli (*Capsicum annuum*).

R gene	Name of the Virus	References
*pvr1*	Potato Virus Y, Pepper mottle virus	[90,91]
*pvr2*	Potato Virus Y	[91,92]
*pvr3*	Pepper mottle virus	[93,94]
*Pvr4*	Pepper mottle virus	[95,96]
*pvr5*	Pepper vein mottle virus	[94]
*pvr6*	Pepper vein mottle virus	[31,97]
*Pvr7*	Pepper mottle virus	[96]
*pepy-1*	Pepper yellow leaf curl Indonesia virus and Pepper yellow leaf curl Aceh virus	[98]

**Table 6:** Reported resistance genes and quantitative trait loci (QTLs) identified against plant viruses in cucurbits.

Host Plant	Group	R Gene	Name of the Virus	References
*Cucumis melo*	Acidulus	*Wmv1551* *Wmr*	Watermelon mosaic virus	[99,100]
		*Prv2* *Prv1*	Papaya ringspot virus	[101,102,103]
	Acidulus	*Zym-1 to Zym-3*	Zuchhini yellow mosaic virus	[104,105,106]
	Conomon, Conomon	*cmv1* *cmv1*, *cmqw3.1*, *cmqw10.1* *Creb-2*	Cucumber mosaic virus	[107,108,109,110,111]
		*Cvy-1* *cvy-2* *Cvy-3*	Cucumber vein yellowing virus	[112]
		*bgm-1*, *Bgm-2*, *Tolcndv*	Tomato leaf curl New Delhi virus	[113]
	Makuwa	*cgmmv-1*, *cgmmv-2*	Cucumber green mottle mosaic virus	[114]
*Cucumis sativus*		*zym-1*	Zuchhini yellow mosaic virus	[115,116]
		*Wmv* *wmv-2*, *wmv-3*	Watermelon mosaic virus	[117,118]
		*prsv* *Prsv-2* *prsv-1*	Papaya ringspot virus	[119,120,121,122]
		*cmv6.1*	Cucumber mosaic virus	[123]
		*Swf-1*, *Swf-2*, *Swf-3 and Swf-4*	Melon yellow spot virus	[124,125]
		*cysdv5.1*	Cucurbit yellow stunting disorder virus	[126]
		*Cscys-1*	Cucumber vein yellowing virus	[127]
*Cucurbita moschata*		*Zym-0*, *zym-4*, *Zym-1*, *Zym-2*, *Zym-3* *zym-6*	Zucchini yellow mosaic virus	[128,129,130,131]
		*Wmv*	Watermelon mosaic virus	[128,132,133]
		*prv*	Papaya ringspot virus	[128,134]
		*cmv*	Cucumber mosaic virus	[128,132]
*Luffa cylindrica*		RG*CLc28*	Tomato leaf curl New Delhi virus	[135]
*Cucurbita pepo*		*QtlZYMV-02* *QtlZYMV-04* *QtlZYMV-08* *QtlZYMV-20*	Zucchini yellow mosaic virus	[136]
*Momordica charantia*		*qYMD.pau_3.1* *q.YMD.pau_4.1* *qYMD.pau_5.1*	Virus complex	[137]

**Table 7:** Reported resistance genes against plant viruses in pea (*Pisum sativum*).

R Gene	Name of the Virus	References
*sbm1*, *sbm2*, *sbm3*, *sbm4*	Pea seedborne mosaic virus	[30,138,139]

**Table 8:** Reported resistance genes against plant viruses in common bean (*Phaseolus vulgaris*).

R Gene	Name of the Virus	Reference
*I*	Bean common mosaic virus	[140]
*bc-1*, *bc-2*, *bc-3*, *bc-4*, *bc-u^d^*	Bean common mosaic virus	[141]

## Data Availability

Not applicable.
